# Flying the RNA Nest: *Drosophila* Reveals Novel Insights into the Transcriptome Dynamics of Early Development

**DOI:** 10.3390/jdb6010005

**Published:** 2018-03-07

**Authors:** Fabio Alexis Lefebvre, Éric Lécuyer

**Affiliations:** 1 Institut de Recherches Cliniques de Montréal (IRCM), Montréal, QC H2W 1R7, Canada; fabioalexis.lefebvre@ircm.qc.ca; 2 Département de Biochimie, Université de Montréal, Montréal, QC H3T 1J4, Canada; 3 Division of Experimental Medicine, McGill University, Montréal, QC H3A 0G4, Canada; 4 IRCM, RNA Biology Laboratory, 110 Avenue des Pins, Ouest, Montréal, QC H2W 1R7, Canada

**Keywords:** embryogenesis, *Drosophila*, development, RNA, transcriptomics, RNA, zygotic genome activation, posttranscriptional regulation, maternal-to-zygotic transition, epigenetics

## Abstract

Early development is punctuated by a series of pervasive and fast paced transitions. These events reshape a differentiated oocyte into a totipotent embryo and allow it to gradually mount a genetic program of its own, thereby framing a new organism. Specifically, developmental transitions that ensure the maternal to embryonic control of developmental events entail a deep remodeling of transcriptional and transcriptomic landscapes. *Drosophila* provides an elegant and genetically tractable system to investigate these conserved changes at a dazzling developmental pace. Here, we review recent studies applying emerging technologies such as ribosome profiling, in situ Hi-C chromatin probing and live embryo RNA imaging to investigate the transcriptional dynamics at play during *Drosophila* embryogenesis. In light of this new literature, we revisit the main models of zygotic genome activation (ZGA). We also review the contributions played by zygotic transcription in shaping embryogenesis and explore emerging concepts of processes such as transcriptional bursting and transcriptional memory.

## 1. Introduction

**Figure 1:**
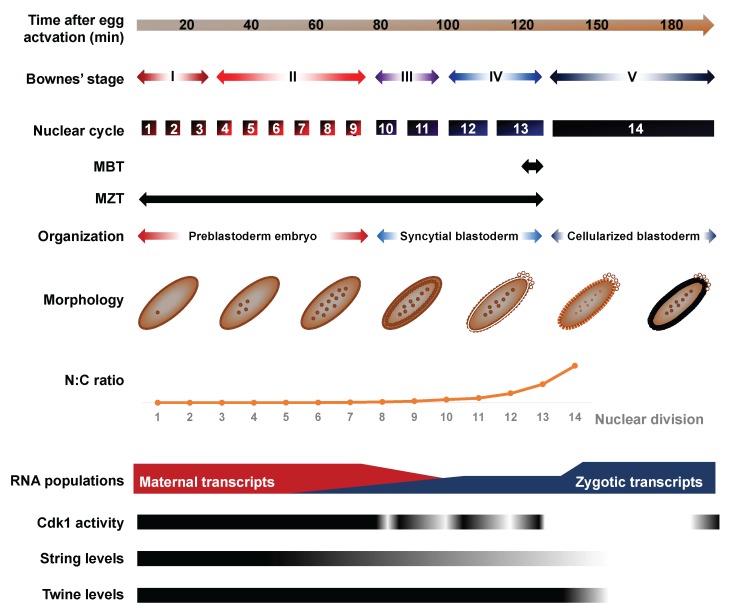
Overview of morphologic and transcriptomic features of early embryonic development. Comparative timescale of Bownes’ stages, nuclear divisions and histological organization as of function of time after egg activation. The nucleocytoplasmic (N:C) ratio increases rapidly during the cleavage cycles, driven by fast-paced mitotic cycles in absence of cytokinesis and growth of the syncytium. The transcriptomic landscape that prevails during the first embryonic cycles reflects maternally-deposited transcripts encoded in ovarian nurse cells and deposited in the oocyte before egg activation. Zygotic genome activation (ZGA) begins in late preblastoderm embryos and leads to the progressive accumulation of zygotic transcripts. Concomitantly, a large fraction of maternally deposited RNAs undergo targeted degradation.

Early development unfolds through exquisitely coordinated and deeply conserved biological transitions. Fertilization entails the remodeling of a differentiated oocyte into a totipotent embryo, which involves profound genomic, transcriptomic and proteomic re-organization [1]. Early embryos execute a dazzling proliferative push driven by maternally provided gene products to increase cell number. The rapid pace of these early divisions, termed cleavage cycles, is achieved through copious supplies of maternal cyclins, an abbreviated DNA replication execution and the absence of active growth and mitotic checkpoints [2]. In most systems, zygotic nuclei remain transcriptionally silent during this period. As embryogenesis proceeds, cell cycle duration progressively lengthens, reflecting the gradual emergence of the DNA replication checkpoint and the increasing destabilization of maternal cyclins [3] (Figure 1). Interphase lengthening broadly coincides with progressive zygotic genome activation (ZGA), i.e., de novo expression of robust populations of functional transcripts [4,5,6]. New zygotic products gradually take over the pool of maternal RNAs, which undergo progressive clearance through regulated degradation mechanisms [7,8,9] (Figure 1). The juxtaposition of ZGA and maternal clearance gradually remodels the transcriptome, a process termed the maternal-to-zygotic transition (MZT). The MZT ends at a key developmental time point called the midblastula transition (MBT), which typically involves a dramatic cascade of anatomical and physiological changes that are dependent on zygotic transcription.

*Drosophila* and many arthropods display a facultative parthenogenetic mode of reproduction, meaning that egg activation can take place independently of sperm entry, although this rarely occurs. The egg-to-embryo transition is triggered by changes in pressure and osmotic balance as the mature oocyte exits through the uterus [10,11]. Egg activation involves the completion of meiosis and the initiation of fast-paced mitotic divisions, thereby setting the onset of embryogenesis. In *Drosophila*, the first rounds of nuclear divisions arise every 9 min, leading to the formation of 6000 nuclei in only 2 h [2] (Foe and Alberts, 1983). To facilitate the rapid pace of these divisions and the synchronization of mitotic entry, nuclear cycles (NC) take place within a single large syncytial cell [12,13] (Figure 1). The first syncytial divisions are metasynchronous, and proceed under maternal control, while zygotic nuclei remain largely transcriptionally quiescent [14,15].

During the interphase of NC8 and NC9, nuclei start migrating to the cortical periphery, forming a syncytial blastoderm embryo [16]. This process coincides with gradual interphase lengthening, reflecting increasing long periods of Cyclin-dependent kinase 1 (Cdk1) inhibition. With its cyclin partners, Cdk1 acts as the chief regulator of cell cycle progression through the phosphorylation of a wide range of protein targets, which notably mediate S phase initiation, spindle assembly and sister chromatid alignment [2]. Cdk1 inhibition expands as the cytoplasmic pool of maternal cyclins, notably String and Twine, is progressively depleted and as the DNA replication checkpoints emerge (Figure 1). In hiatus during the first cycles, the checkpoint safeguards genome integrity by preventing mitotic entry when single-stranded (ss)DNA is sensed, underlying incomplete replication or extensive damage [17,18]. When the MBT takes place at NC13, interphase duration suddenly triples as mitotic synchrony is lost and cortical nuclei secede from the syncytium to form a well-defined primordial epithelium, a process termed cellularization [2]. En masse zygotic transcription ensues in cells now endowed with motility and a susceptibility for apoptosis—new-found attributes that will play crucial roles in gastrulation, neurulation and organogenesis [19,20].

Evolutionarily conserved features of the MZT have been well reviewed by Tadros and Lipsitz (2009) and more recently by Langley and colleagues (2014). This exciting area of developmental biology has further expanded over the last few years, along with our understanding of the complex cross-talks resulting in the interrelated emergence of ZGA, checkpoint activation and cellularization. Indeed, the deployment of disruptive technologies to track translation, probe chromosome conformation and image single RNA molecules in *Drosophila* have revealed new insights into the organization of the transcriptome during embryogenesis. Here, we focus on key findings established in recent years that paint an increasingly resolved picture of transcriptome dynamics during early development. We first review the changes in transcriptomic landscapes that take place as maternally-deposited transcripts are selectively localized, translated and degraded. We discuss the different models proposed to regulate the onset of zygotic transcription and focus on the remodeling of chromatin during early embryogenesis. We then review the roles played by zygotic transcription in promoting cellularization, maternal clearance and replication checkpoint activation. Finally, we outline novel properties of zygotic transcription revealed by real-time, in vivo imaging, including transcriptional bursting and transcriptional memory.

## 2. Deposition, Localization, Translation and Clearance of Maternal Transcripts

Early *Drosophila* development is driven by maternal proteins and RNA transcripts synthesized by multiploid nurse cells and deposited in the egg during oogenesis [21,22]. Long before fertilization, structural and biosynthetic factors such as ribosomal and glycolytic proteins are endowed in the oocyte along with their corresponding mRNAs [23]. These factors will direct rapid mitotic cycling and sustain DNA replication in early embryogenesis, while zygotic nuclei remain transcriptionally quiescent. Maternal deposition is widespread: up to 65% of all annotated *Drosophila* mRNAs can be detected during the first syncytial cycles [24,25,26]. Among these products, a set of maternal mRNAs acquire spatially-resolved localization in the oocyte through a series of symmetry breaking events during ovarian development. After egg activation, the asymmetric distributions of these transcripts, notably *bicoid*, *oskar* and *gurken*, defines anteroposterior and dorsoventral axes, which will later determine the body plan of the developing fly [27,28,29]. Aside from these classically-defined examples, recent large-scale *fluorescent* in situ *hybridization* (FISH) surveys have demonstrated that subellular localization is a pervasive feature of the *Drosophila* transcriptome. Indeed, detailed profiling of RNA expression/localization features in fly oocytes and embryos, as tabulated in the Fly-FISH (http://fly-fish.ccbr.utoronto.ca/) and Dresden Ovary Table/DOT (http://tomancak-srv1.mpi-cbg.de/DOT/main.html) database repositories, revealed that the vast majority of mRNAs and long non-coding RNAs are localized in a striking array of distribution patterns [24,30]. While these studies underline the dynamic localization properties of both maternal and zygotic RNA populations, the underlying regulatory mechanisms and functions for the most part remain to be characterized.

**Figure 2:**
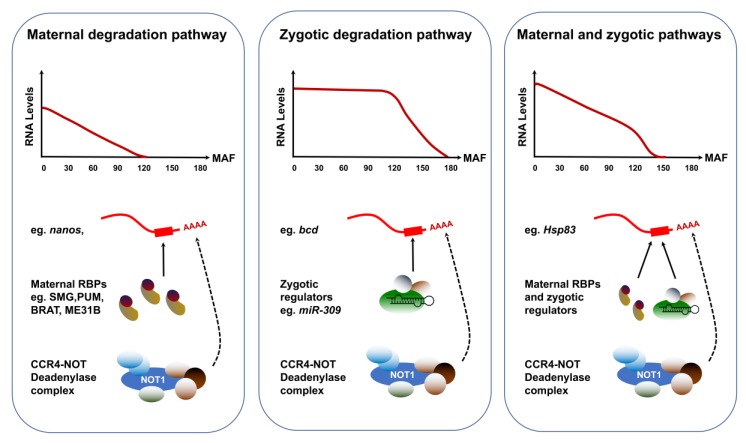
Alternative degradation profiles of maternally-deposited transcripts. The clearance of maternally-deposited transcripts can proceed through a strictly maternal pathway, a strictly zygotic pathway or a combination of both maternal and zygotic effectors. Transcripts strictly targeted by the maternal pathway, such as *nanos* (*nos*) display identical dynamics in fertilized and activated eggs. The maternal RNA-binding proteins Smaug, Pumilio, Brat and/or ME31B selectively interact with these RNAs through a consensus motif and recruit the CCR4/POP2/NOT deadenylase complex to initiate their degradation. Transcripts targeted through the zygotic pathway include *bicoid* (*bcd*) and their degradation depends on the ZGA. Transcripts targeted through both maternal and zygotic degradation pathways include *Hsp83* and their clearance relies on the activity of both maternally deposited and zygotically encoded factors. MAF: Minutes after fertilization.

The transcriptomic landscape is highly dynamic during the cleavage cycles, as several RNA degradation pathways operate to selectively remove large sets of deposited transcripts. Indeed, a study using chromosomal deletions to track the post-transcriptional dynamics of maternal and zygotic transcripts has shown that approximately 35% of maternally-deposited mRNAs are cleared out by the MBT [31]. Comparisons of degradation dynamics in unfertilized and fertilized embryos have revealed the prevalence of at least two pathways. The early onset maternal pathway operates independently of ZGA and accounts for the destabilization of approximately 20% of all mRNAs [8] (Figure 2). This maternal degradation program reflects the coordinated activity of RNA-binding proteins (RBPs) that associate with specific subsets of maternal transcripts and recruit the CCR4-NOT deadenylase complex to initiate their degradation [32]. Maternal clearance is a highly-coordinated process determined by the interplay of *cis*-acting motifs, generally found in the 3′UTR of the target transcripts, and RBPs that adopt tightly regulated spatial and temporal distributions. Indeed, mRNAs encoding the RBP Smaug (SMG) form an anterior-to-posterior gradient in the oocyte and are translationally regulated at egg activation [33]. SMG activity peaks at NC10, enacting an elegant spatial and temporal regulation of maternal clearance [32,33,34]. In addition, three RBPs, Pumilio (PUM), Brain tumor (BRAT) and ME31B have more recently been shown to have non-overlapping roles in mediating the decay of hundreds of maternal transcripts [35,36].

**Figure 3:**
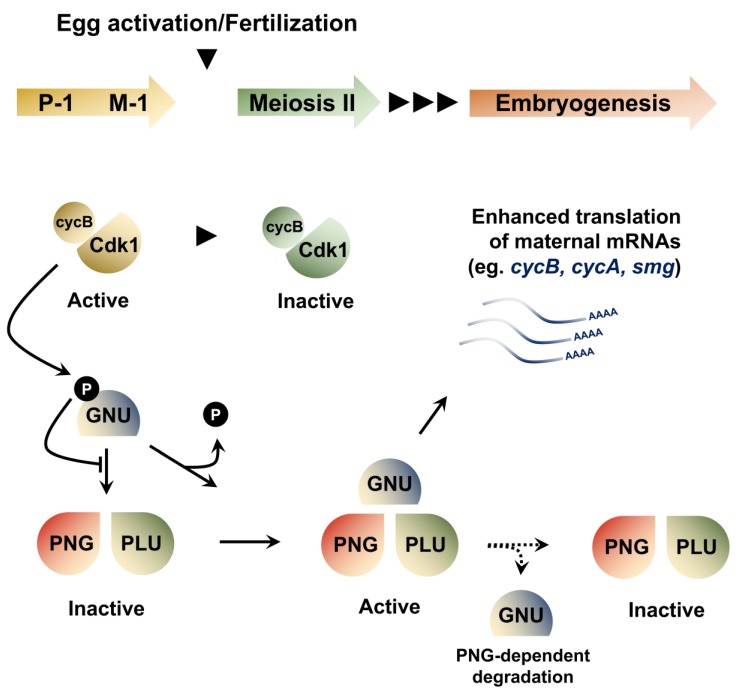
Developmental regulation of the PNG kinase coupled to cell cycle progression. In mature oocytes arrested at metaphase of meiosis I, cyclinB-CDK1 dependent phospohorylation of GNU exerts an inhibition of PNG complex assembly and activation. The completion of meiosis that follows egg activation results in CDK1 inactivation, prompting the dephosphorylation of GNU. In meiosis II, the accumulation of dephosphorylated GNU proteins leads to the spontaneous assembly of an active PNG kinase complex, consisting of GNU, PNG and PLU. The PNG kinase regulates the translation of hundreds of maternal mRNAs, including *cycB* and and *smg*. In addition, GNU protein degradation is promoted by PNG activity, enacting a negative feedback loop that restricts the activity of the complex to the temporal context of early embryonic development.

Strong evidence in zebrafish, *Xenopus* and mouse has long suggested that some of the zygotic actors involved in maternal clearance are microRNAs (miRNAs) [37]. Small-interfering RNAs are versatile regulators that can destabilize vast pools of mRNA targets [37,38]. In support of this model, SMG is required for the zygotic expression of the miR-309 family [39]. Among the miRNAs expressed during the MZT, over 70 exhibit compromised levels in *smg* mutants. In addition, Argonaute-1, a key component of the miRNA-induced silencing complex, showed decreased levels in *smg* mutants. Furthermore, the clearance of predicted maternal targets of SMG-dependent miRNAs is hampered in *smg* mutants, suggesting that these small RNAs contribute to the zygotic component of maternal mRNA degradation [39,40]. Because of the specificity and diversity of clearance pathways effective during the MZT, the degradation kinetics of maternal transcripts exhibit striking diversity (Figure 3). The abundance of transcripts strictly targeted by maternal degradation RBPs, such as *nanos* (*nos*), starts declining linearly after egg activation. By contrast, targets of zygotic degradation effectors such as *bicoid* (*bcd*) are untouched during the first cycles and their removal begins after ZGA. Finally, transcripts targeted through both maternal and zygotic pathways include *Hsp83* and adopt highly specific degradation kinetics that reflect the contributions and levels of both maternal and zygotic factors.

The execution of maternal clearance is tightly coupled to translational regulation of RBPs that mediate mRNA degradation. Indeed, the levels of SMG depend on the PAN GU (PNG) Ser/Thr kinase complex, a key translational regulator activated at the oocyte-to-embryo transition. The PNG complex is involved in reprogramming the proteome as the egg becomes an embryo by regulating the translation of hundreds of maternal mRNAs (Figure 3). Its notable targets include *cyclin B* (*cycB*), which encodes a key regulator of the embryonic cell cycle [41,42,43]. The complex consists of three proteins, PNG, GNU and PLU. Mutations in any of these genes leads to a profound decrease in Cyclin B protein levels, without affecting the corresponding mRNA levels. A recent study revealed a feedback loop involved in the regulation of PNG activity by the Cyclin B/CDK1 complex at the oocyte-to-embryo transition [44]. In mature oocytes, PNG activity is kept in check through inhibitory CyclinB/CDK1-dependent phosphorylation of the GNU activating subunit, preventing its association with PNG. Meiosis completion coincides with a decrease in CyclinB/CDK1 activity, leading to GNU dephosphorylation, which can then activate the PNG kinase, unleashing its widespread translational activation. However, sustained PNG kinase activity leads to a decrease in GNU levels, providing a mechanism to end PNG kinase function after egg activation and restrict its activity to the oocyte-to-embryo transition period (Figure 3).

In another study, ribosome profiling performed on mature oocytes and activated eggs derived from *png* mutant mothers revealed compromised translational efficiencies in nearly 1000 mRNAs [45]. Surprisingly, it was found that translational upregulation poorly reflects on protein levels in fertilized eggs, leading the authors to propose the existence of a “resetting” process in which enhanced translation is counterbalanced by proteasomal degradation, perhaps enabling the removal of proteins bearing oocyte-specific posttranslational modifications. Interestingly, this model is reminiscent of the well-characterized MZT phenomenon that takes place later in embryogenesis, as many maternal transcripts are degraded and subsequently expressed de novo from the zygotic genome [22].

Overall, spatiotemporal regulation enacted through mRNA localization and translation control are key features of embryonic transitions. In the absence of large-scale zygotic transcription, maternally deposited mRNAs and their regulated translation drive the developmental program of early embryogenesis. These maternal transcripts are then selectively removed, a process relying on the recognition of *cis*-acting motifs by maternal RBPs that are tightly regulated in time and space. As zygotic transcription emerges, a second phase of maternal clearance unfolds through the activity of zygotically expressed determinants (e.g., miRNAs and RBPs).

## 3. Models of Zygotic Genome Activation

**Figure 4:**
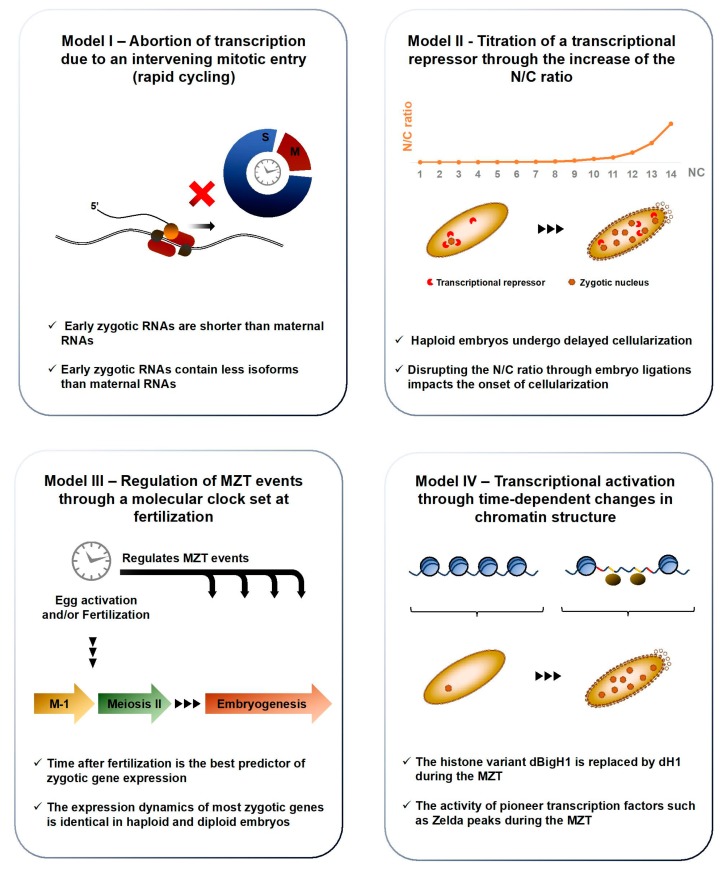
Models of zygotic genome activation. Several models have been proposed to contribute to ZGA. Each is supported by a set of empirical evidence (outlined under each cartoon) and these different propositions likely contribute synergistically to the emergence of zygotic transcription. Model I posits that early zygotic transcription is restricted due to the short duration of interphases during early embryogenesis, effectively preventing the complete transcription of long genes. Model II stipulates that zygotic transcription is prohibited during early embryogenesis due to the abundance of a maternally-inherited transcriptional repressor. The titration of this repressor against the increasing mass of zygotic nuclei would progressively lead to transcriptional competence. Model III proposes that egg activation sets in a molecular clock, which times key events of the MZT, including ZGA. Model IV postulates that chromatin is kept in a state that precludes transcription during early embryogenesis and is progressively remodeled through active changes in its composition to promote gene expression.

As maternal clearance takes place during the cleavage cycles, the transcriptomic landscape is remodeled and new populations of transcripts arise upon activation of the zygotic genome. The mechanisms behind ZGA onset are poorly understood and remain an outstanding question in developmental biology, although several models have been proposed [7] (Figure 4). Seminal work involving the injection of plasmids in *Xenopus* embryos showed that early transcription is possible prior to ZGA [46,47]. However, the expression of plasmid DNA is rapidly silenced and resumes at the normal timing of transcription initiation. This observation suggests that the early zygotic genome is transcriptionally competent and actively repressed during the cleavage cycles. Moreover, ZGA is a gradual process and delineating its onset has remained challenging. Indeed, the emergence of the first zygotic transcripts has long been associated with the acquisition of the syncytial blastoderm morphology, at NC8. However, the detection of a small subset of zygotic genes before NC7, including the transcription factor *engrailed* (*en*), has recently been reported in preblastoderm embryos, with key implications in establishing the synchrony of early mitotic cycles [48]. Regardless of the exact onset of their expression, the first zygotic products display a conserved tendency to encode few exons and their length is shorter than maternally provided mRNAs [5]. In line with this observation, one hypothesis is that transcription is systematically attempted during the first cleavage cycles but that nascent transcripts are largely aborted, due to excessively fast cycling [7,15,46,49,50,51]. Hence, zygotic genes may be shorter than populations contributed maternally because Pol II can complete their transcription prior to an intervening mitotic entry [5,6].

One long-standing model posits that ZGA is triggered once a critical value of nucleocytoplasmic (N/C) ratio is breached in the syncytium [2,4,15,52,53]. Indeed, the ratio of nuclei to cytoplasm increases rapidly during early embryogenesis, as the nuclear count expands exponentially and the volume of syncytial cytoplasm is kept constant due to growth inhibition. Tampering with this ratio by constricting embryos and compounding or reducing chromosome size impacts the number of syncytial divisions and the onset of cellularization [53]. It is thought that the increasing DNA mass titrates a maternal factor required to sustain fast-paced proliferation, until its concentration has decreased sufficiently to terminate the early cell cycle program. Similarly, a transcriptional repressor inherited maternally could be embedded in chromatin and diluted as the mass of zygotic DNA increases. After a certain N/C threshold is breached, the concentration of this repressor would have sufficiently decreased to allow zygotic transcription to take place. One enduring rival of the N/C proposition has been called the “molecular clock” model; it proposes that egg activation sets a chronological countdown that times MZT events, including ZGA [2,15]. A study comparing the onset of transcription in haploid and diploid embryos, which differ markedly in N/C ratio, found little difference in the expression dynamics of most zygotic genes, suggesting that the molecular clock model is the best overall predictor of ZGA onset in *Drosophila*. Interestingly, the authors identified a small subset of genes regulated in a N/C-dependent manner, suggesting that both models prevail, albeit at distinct loci and frequencies [54]. Nevertheless, the molecular identity that senses the clock to activate zygotic transcription independently of the N/C ratio remains elusive.

**Figure 5:**
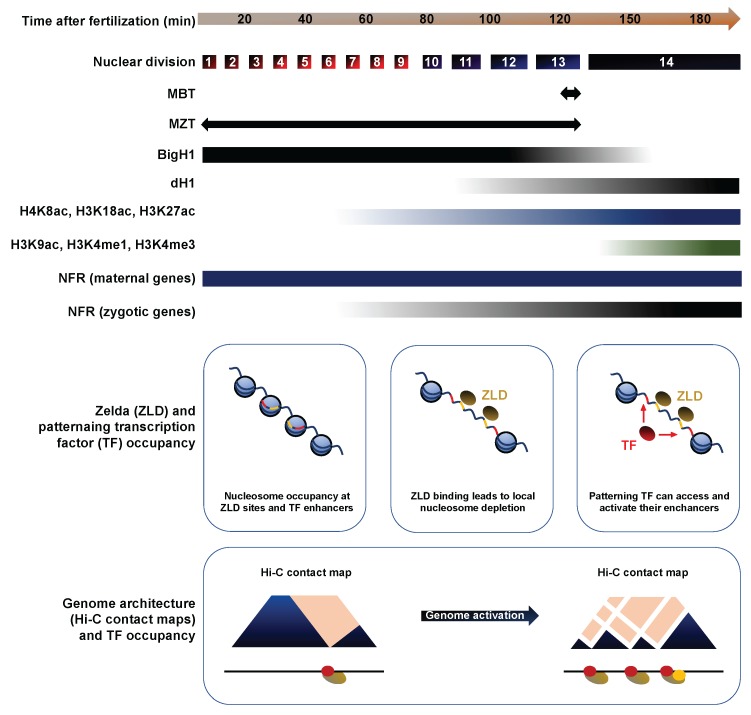
Developmental regulation of chromatin landscapes and genomic architecture. The histone H1 variant BigH1 is a constitutive chromatin component in the germline and in fertilized embryos until the MBT. The acetylation marks H4K8ac, H3K18ac and H3K27ac appear at the ZGA and scale up with the prevalence of zygotic transcription in syncytial embryos. By contrast, H3K9ac and H3K4me1/3 emerge around the MBT. Nucleosome-free regions (NFR) are found upstream of maternally-deposited genes throughout embryonic development but their appearance upstream of zygotic genes is concomitant with their transcription. Endowed with pioneer transcription factor activity, the binding of Zelda (ZLD) to its consensus sequence leads to local nucleosome depletion around NC10, exposing surrounding enhancers to promote the recruitment of patterning transcription factors by NC14. Hi-C data shows that chromatin is poorly organized prior to NC10. The emergence of intricate long-range interactions emerges after ZGA, by NC14.

Hence, diverse mechanisms have been proposed to account for ZGA onset, each of which is supported by empirical evidence [7]. How these different propositions converge to enact a precise execution of genome activation remains elusive. The key to ZGA regulation might lie in the reorganization of chromatin. Interestingly, a rapidly expanding literature is helping to define how chromatin states relate to transcription in early embryos (Figure 4 and Figure 5).

## 4. Chromatin Rearrangements and Zygotic Genome Activation

Histone proteins are fundamental components of chromatin and developmentally-regulated changes in their expression could have profound impacts on genome activation. Indeed, most metazoans express tissue-specific variants of the linker histone H1 and the somatic H1 is often replaced by a developmental variant during early embryogenesis [55,56]. In a recent article, Pérez-Montero et al. identified the first H1 variant in *Drosophila*, called dBigH1, and demonstrate its involvement in ZGA regulation [57]. Ubiquitous in preblastoderm and syncytial blastoderm embryos, dBigH1 is progressively replaced by dH1 in somatic cells at the MBT, except in primordial germ cells (PGCs), in which it is retained well after gastrulation (Figure 5). *BigH1^00^* mutants exhibit high embryonic lethality and a range of developmental defects, including altered nuclear distributions and highly asynchronous divisions. Interestingly, ChIP showed that Pol II is recruited to chromatin earlier in *BigH1^00^* mutants than in *wt* embryos. In addition, zygotic mRNAs were more abundant in *BigH1^00^* mutants than in *wt* embryos 2h after fertilization. Together, these results show that BigH1, a novel *Drosophila* variant of the linker histone, regulates ZGA onset and is removed from chromatin prior to the MBT.

The histone code refers to a set of posttranslational modifications that modulate chromatin compaction and the accessibility of DNA elements [58]. Changes in this epigenetic landscape likely contribute to the emergence of zygotic transcription. In a recent study, Li et al. investigated the genome-wide distribution of nine histone marks using staged embryo collections at NC8, NC12 and NC14. Prior to ZGA (NC8), chromatin exists in a relatively simple state, lacking histone methylation (me) and displaying low levels of histone acetylation (ac) or nucleosome free regions (NFRs), a hallmark of transcriptional activity [59]. The acetylation marks H4K8ac, H3K18ac and H3K27ac appeared along with transcription by NC12. By contrast, H3K9ac and the methylation marks H3K4me1, H3K4me3, H3K27me3 and H3K36me3 are only apparent after the MBT, at NC14 (Figure 5). As reported by previous studies, NFRs are prevalent upstream of maternally deposited genes even in absence of zygotic transcription, suggesting that nucleosome depletion is stable across development [60]. To identify putative blastoderm enhancers, Li et al. calculated the cumulative binding of 16 early transcription factors and examined the sites showing the highest cumulative occupation, excluding known promoters and coding regions. They found that putative enhancers display relatively high nucleosome density at NC8, with the appearance of acetylation marks by NC12 and H3K4me1 by early NC14, whereas the repressive mark H3K27me3 only spreads in surrounding regions by late NC14 (Figure 5).

The factors that act between NC8 and NC14 to deplete enhancer-associated nucleosomes represent major instigators of ZGA. Transcription factors (TFs) that can recognize their binding sites in a closed chromatin context to promote chromatin remodeling are termed “pioneer TFs”. Several studies have identified such pioneer activity for the Zn-finger transcription factor Zelda/Vielfaltig (ZLD/VFL), a master regulator of early zygotic gene expression [61,62]. Indeed, ZLD is detected by NC2 in syncytial embryos and its binding displays a striking correlation with the timing and magnitude of early zygotic transcription [63]. ZLD has been shown to prime enhancers by lowering the nucleosome barrier sufficiently to promote the accession of specific binding motifs by their associated TFs [64]. These observations suggest that ZLD may act as a global genome activator in *Drosophila*, like Nanog, Pouf5f3 and SoxB1 in vertebrates [65,66]. Indeed, Li et al. found that nearly all the putative blastoderm enhancers identified through cumulative TF binding at NC14 are already bound by ZLD at NC8. Moreover, the ZLD consensus motif CAGGTAG was the single most enriched sequence associated to the early enhancer marks H3K27ac, H3K18ac and H4K8ac. Finally, H3K4me1 was lost and H3K18ac strongly compromised at ZLD-bound regions in embryos obtained from *zld^-^* germline clones. Overall, Li et al. show that histone marks are depleted during the first cleavage cycles and emerge between NC8 and NC14. Importantly, ZLD is a pioneer TF of the MZT: it can bind its genomic sites in condensed chromatin at NC8 and promote the recruitment of other factors that carry out profound chromatin remodeling at the MBT [67,68].

In addition to histone modifications, the three-dimensional folding of chromosomes can bring distant genomic loci in close physical proximity, with profound impacts on gene expression [69]. Topologically associated domains (TADs) are regions of high contact probabilities that display significant insulation from neighboring loci, enabling enhancer-promoter contacts and the coordination of gene expression programs [70,71]. Chromosome conformation capture (3C), its adaptations (4C and 5C) and the recent genome-wide variant Hi-C can reveal TADs with increasing resolution. They have been optimized in *Drosophila* embryos, enabling investigations into the developmental implications of genome architecture [72,73,74,75]. Recently, *Hug* et al. performed Hi-C at time-points surrounding the ZGA to determine when chromatin architecture is established during development and how its emergence relates to the onset of zygotic transcription [76]. NC8 embryos display poorly organized chromatin, exhibiting broadly uniform contact probabilities through large genomic distances. By contrast, NC13, NC14 and gastrula embryos revealed increasingly strong enrichments of chromatin associations within TADs and sharply declining contact frequencies with the loci surrounding TADs. This picture suggests that chromatin architecture is rapidly remodeled from an unordered state in preblastoderm embryos to a structured organization by NC14 (Figure 5). These boundaries are tightly maintained in later-stage embryos and in Kc167 cells, consistent with highly stable TAD boundaries described in other models (Dixon et al., 2012).

ChIP-seq revealed a strong dose-dependent correlation between Pol II occupancy and TAD boundary-like regions, especially at housekeeping genes and across developmental stages. Analysis of the early zygotic *Bsg25*/*Elba3* locus, which is switched off before gastrulation, showed that loss of Pol II occupancy at NC14 coincides with the loss of its boundary-like structure [77]. Together, these results suggest that Pol II binding contributes to chromatin conformation reorganization. To test the role of transcription, Hug et al. injected NC8 embryos with the Pol II inhibitors α-amanitin and triptolide before performing Hi-C to examine chromatin architecture at the MBT. Inter-TAD insulation was compromised at NC14, as well as the co-localization of housekeeping gene boundaries, although extensive long-distance contacts were still prevalent independent of transcription. ZLD occupancy showed striking correlations with TAD boundaries by NC12, hinting at a potential role in their establishment. To explore this hypothesis, Hug et al. performed in situ Hi-C on NC14 *zld^-^* embryos, which revealed a loss of insulation of TAD-boundaries at strong ZLD sites, especially at boundaries established in early cycles. Collectively, Hug et al. provide strong evidence that the establishment of long-range interactions broadly coincide with ZGA. Although transcription is not required for the emergence of chromatin conformation, loci transcribed early act as nucleation sites and contribute to the establishment of TAD boundaries. Similarly, ZLD binding contribute significantly to TAD boundary insulation, consistent with ZLD roles as global activator of the zygotic genome.

In brief, ZGA coincides with a profound reorganization of chromatin. Prior to NC8, chromatin exhibits a simple and disorganized state, with few histone modifications, NFRs or TADs. The germline-specific histone variant dBigH1 is embedded in chromatin, possibly contributing to its transcriptional silencing. Through pioneer TF activity, factors such as ZLD disrupt the nucleosome barrier between NC8 and NC12 to expose zygotic enhancers and promote the transcription of their target genes. Concomitantly, the activating histone marks H4K8ac, H3K18ac and H3K27ac appear and TADs emerge. By the MBT, dBigH1 has been replaced by histone H1, long-range interactions have gained complexity and stability and the histone marks H3K4me1, H3K4me3, H3K27me3 and H3K36me3 are established.

## 5. ZGA as a Driver of Embryonic Development

After its emergence, zygotic transcription becomes a major driver of embryonic development. Its contribution is twofold: zygotic products directly enact important functions, notably transcription factors that reshape the developmental program and miRNAs that contribute to maternal clearance. In addition, the process of transcription itself seems to mediate changes in the biology of the embryo. Indeed, active transcription can expose ssDNA and may cause replication stalling when facing a replication fork. These processes have recently been linked to the activation of the DNA replication checkpoint before the MBT. Indeed, studies taking advantage of mutants with impaired ZGA have revealed that transcription contributes to maternal clearance and determines the onset of cellularization and replication checkpoint activation.

**Figure 6:**
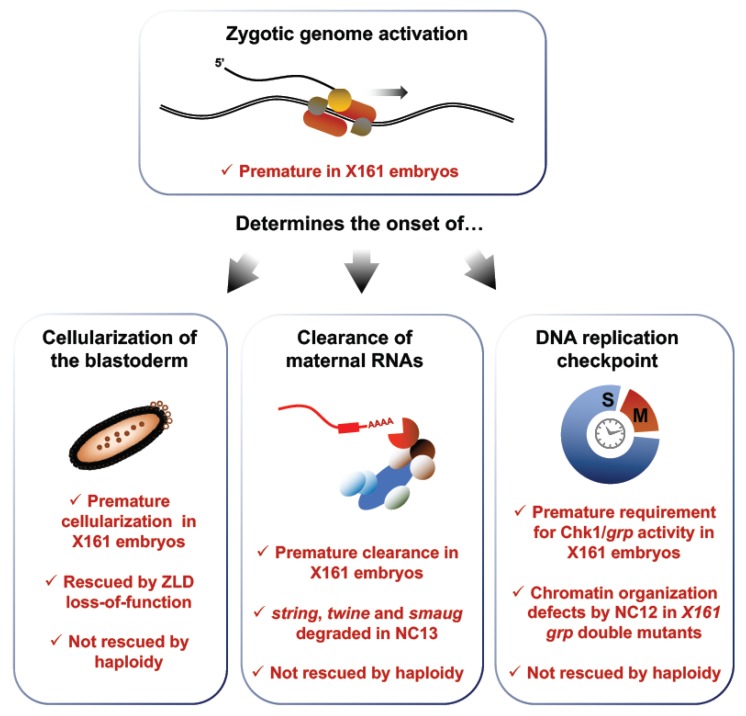
Zygotic genome activation times the onset of the DNA replication checkpoint, maternal clearance and cellularization. X161 mutants display premature zygotic transcription due to a point mutation in the *RNPII215* gene, which encodes a subunit of Pol II. This disruption leads to premature cellularization, which is rescued by altering transcription in X161 *zld* double mutants. Precocious transcription also leads to an early deployment of maternal clearance, exemplified by the premature degradation of the maternal transcripts *string*, *twine* and *smaug*. In addition, early ZGA leads to a premature activation of the replication checkpoint, as inferred from a precipitate requirement for Chk1/grp activity. None of these phenotypes are rescued in haploid X161 embryos, which exhibit a decreased N/C ratio, meaning that the N/C ratio does not act upstream of transcription activation in the regulation of cellularization, maternal clearance and checkpoint activation.

Sung et al. characterized a fly model exhibiting a point mutation in the 3′ untranslated region (3′UTR) of the *RNPII215* gene, which encodes the large subunit of Pol II [78]. These mutants, termed X161 embryos, undergo premature zygotic transcription onset, providing an appealing model to investigate the complex relationships between zygotic transcription and other key events of the MBT (Figure 6). Interestingly, X161 embryos terminate the syncytial stage after the completion of 12 NC rather than 13 NC, suggesting that interfering with ZGA onset impacts the timing of cellularization. To confirm this observation, Sung et al. considered mutants for the master transcription factor Zelda (ZLD), which fail to transcribe a broad set of early zygotic genes. They found that X161 *zld* double mutants all undergo 13 syncytial NCs, like *wt* embryos. Since ZLD loss-of-function rescues the premature transcription phenotype of X161 mutants, a normal number of syncytial cycles in X161 *zld* double mutants suggests that early transcription causes the precocious cellularization phenotype of X161 single mutants. In addition, Sung et al. used the X161 model to test the contribution of the nucleocytoplasmic ratio on cellularization. Haploid X161 embryos, which present a lowered N/C ratio, underwent only 12 NC, the same number as diploid X161 mutants. This result suggests that the N/C ratio acts independently of ZGA and does not directly regulate the onset of cellularization in *Drosophila*. Together, these experiments provide strong evidence that the onset of zygotic transcription times key events of early embryonic development.

As discussed earlier, maternal clearance is a complex process relying on factors contributed maternally and on the expression of zygotic products. Sung et al. surveyed the levels of three canonical targets of maternal clearance, *string*, *twine* and *smaug* in X161 mutants to monitor the impact of ZGA onset on maternal clearance. They found that the degradation of these maternal transcripts, which starts during the 14th interphase in *wt* embryos, is already well advanced by NC13 in X161 embryos, in agreement with reports of a zygotic contribution to maternal clearance. The authors also found that premature ZGA leads to a precocious requirement for a functional replication checkpoint. Checkpoint activation pauses M phase entry until the completion of DNA replication to safeguard genome integrity. Its emergence is a gradual process completed at the MBT. The Ser/Thr kinase Chk1, encoded by the *Drosophila* gene *grapes* (*grp)*, is a key component of the DNA damage response (DDR), signal transduction cascades that sense DNA lesions to halt mitotic entry [17,79]. Chk1 activity is required for progression through the MBT and its loss leads to genomic instability exemplified by chromatin defects and embryonic lethality. Chk1 activity is dispensable prior to NC13, but necessary around the MBT, when its loss leads to the apparition of genotoxic lesions. Therefore, the requirement for Chk1 activity can be used as a proxy to score the onset of checkpoint activation. Sung et al. found that X161 *grp* double mutants display nuclear envelope and chromatin condensation defects by the 13th interphase, one cycle earlier than *grp* single mutants, supporting a role of transcription in the onset of the DNA replication checkpoint activation.

Interestingly, the nuclear retention of zygotic transcripts has been identified as a new facet of the DDR during early embryogenesis. Indeed, Iampietro et al. showed that syncytial-stage embryos challenged with genotoxic stress undergo extensive nuclear fallout at the MBT, a mechanism of programmed elimination [18]. The authors showed that fallout nuclei display widespread nuclear retention of diverse zygotic transcripts, including histone mRNAs. The nuclear retention of histone mRNAs is linked to a Chk2-mediated phosphorylation of the stem loop binding protein (SLBP), which orchestrates the posttranscriptional processing and nuclear export of histone mRNAs. In turn, the nuclear retention of essential mRNAs such as histones leads to a local depletion of their corresponding proteins in the vicinity of damaged nuclei, promoting their fallout and elimination from the somatic pool. Prior to the establishment of a robust DNA replication checkpoint, the propensity of syncytial embryos to the accumulation of DNA lesion is thus mitigated through a Chk2-mediated nuclear fallout process that relies on the nuclear retention of essential mRNAs. These results reveal a novel role of posttranscriptional transport routes in ensuring genome integrity surveillance during embryogenesis.

*Blythe and Wieschaus* provided further evidence of the interplay between zygotic transcription and replication checkpoint activation. These authors found that checkpoint activation correlates with the amount of DNA engaged by Pol II, independently of the N/C ratio [80,81]. Through ChIP-seq analyses, then found that Pol II distributions are not severely impaired in *grp* mutants, with widespread genomic occupancy at NC12, NC13 and NC14. This result suggests that the transcriptional machinery is in place independently of the functionality of the replication checkpoint. To investigate the links between checkpoint activation and ZGA at the molecular level, the authors took advantage of RPA70, an important effector of the DDR. RPA70 binds stress-induced ssDNA produced when replication is stalled, leading to ATR (*mei-41*) recruitment and checkpoint activation. Assessment of RPA70 occupancy through fluorescent microscopy and ChIP-seq revealed a strong correlation with Pol II binding sites, consistent with the hypothesis that Pol II engagement activates the checkpoint at the MBT. In *zld^-^* mutants, ChIP-seq analyses revealed altered RPA70 occupancy at *zld*-dependent promoters. This result suggests that transcription contributes to checkpoint activation.

To test this hypothesis, the authors attempted to rescue the lethality phenotype associated to mutations in the DDR factor ATR by altering ZGA through different approaches. They showed that most embryos from double *zld mei-41* (ATR) mutants complete cleavage cycles and that many escape the mitotic catastrophe that characterizes *mei-41* mutants. In addition to the *zld^-^* model, they used a heterozygous deficiency in the transcriptional activator Trithorax-like/GAGA (*Trl*), associated to defects in the genomic recruitment of poised Pol II, to interfere with ZGA. They found that *mei-41*; *Df(3L)ED4545/+ (Df(trl)/+)* embryos complete cleavage cycles without a mitotic catastrophe after a slightly lengthened NC13 and eventually yield hatching larvae. In addition, heterozygosity of the *cyclin B* gene (*Df(cycB)/+*), which lengthens NC13 time, effectively suppressed the mitotic catastrophe of *mei-41* mutants. Together, these rescue experiments show that reducing the source of replication stalling by interfering with transcription (in *zld* and *Trl* mutants) and providing more time to allow DNA replication (in *cycB* mutants) can bypass the MBT requirement for a functional replication checkpoint. In conjunction with evidence of RPA70 colocalization with the transcriptional machinery, these results strongly suggest that the replication checkpoint is activated in response to ZGA.

In brief, recent studies have used loss-of-function analyses to reveal the contributions of ZGA to key facets of embryonic development. Mutants exhibiting a premature ZGA undergo early cellularization, promptly enact maternal clearance and acquire a precocious requirement for effectors of the DDR. These effects are independent of the N/C ratio and can be rescued by modulating zygotic transcription. Moreover, effectors of the DDR are recruited to chromatin at Pol II occupied loci after ZGA and interfering with the scope of zygotic transcription can bypass the requirement for a functional checkpoint. In addition, DNA damage elicits a Chk2-dependent clearance of damaged nuclei in syncytial embryos through the nuclear retention of essential mRNAs, providing an elegant mechanism to safeguard genome integrity prior to the establishment of a robust DNA replication checkpoint.

## 6. Emerging Properties of Zygotic Transcription

**Figure 7:**
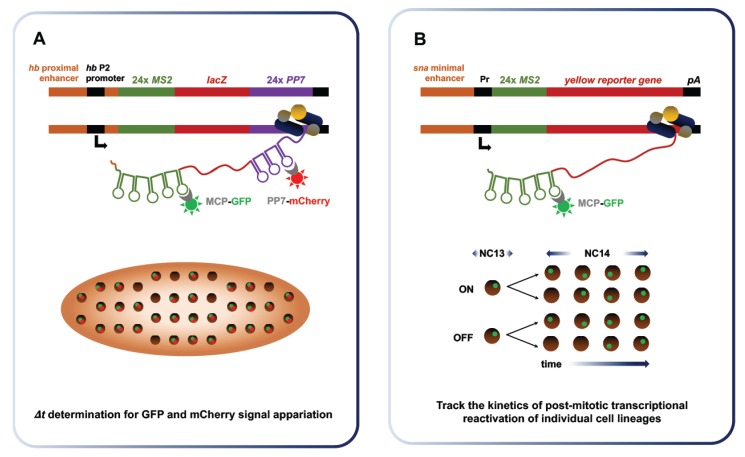
Real-time imaging of zygotic transcription enables the determination of Pol II elongation rate and demonstrates transcriptional memory at NC14. (**A**) Fukaya et al. used a dual fluorescence approach involving the MS2 system to measure Pol II elongation rates at NC13. They integrated a construct encompassing a *lacZ* reporter flanked by 24 *MS2* repeats at its 5′ end and 24 *PP7* repeats at the 3′ end. Its expression in conjunction with the MCP-GFP and mCherry-PP7 coat proteins leads to the emission of dual green and red fluorescence. By measuring the delay between the emission of the green and red signals at the single molecule level, the time required to transcribe the intervening *lacZ* sequence can be determined. This value is then used to calculate the elongation rate of Pol II. (**B**) Ferraro et al. provided evidence of transcriptional memory by monitoring post-mitotic reactivation. Sensitized transgenes were used to obtain sporadic expression of the *yellow* reporter downstream of 24 *MS2* repeats. They tracked expression during NC13 and NC14 in a lineage-specific manner. After mitosis, the authors found that the daughter of nuclei having expressed the reporter during NC13 where four times more likely to re-express it rapidly. This result indicates that transcription prior to mitosis increases the chance and rapidity of re-expression across cell generations, a phenomenon termed transcriptional memory.

While zygotic transcription drives key events of embryonic development, the properties and dynamics of the emerging transcriptional process itself have been challenging to study. The advent of approaches enabling RNA labeling in vivo and in real time has provided a clearer picture of zygotic transcription. Originally developed by Singer and colleagues in yeast, the MS2 system takes advantage of the strong affinity of bacteriophage coat proteins (e.g., MS2, PP7) for specific RNA stem-loops [82]. For imaging purposes, MS2 phage coat protein fused to a fluorescent reporter (e.g., GFP, mCherry) are co-expressed in a transgenic organism along with an RNA fusion that encompasses the target transcript and MS2 stem-loop repeats (Figure 7). Stable tethering of the coat fusion protein allows for durable tracking of the target RNA, which can be expressed in its endogenous regulatory context to recapitulate physiological properties [83]. Over the last decade, several groups have harnessed the power of the MS2 imaging system to study RNA dynamics and localization during *Drosophila* development [84,85,86,87,88,89] This system has notably been used to investigate the dynamics of transcriptional bursting, calculate Pol II elongation rate at the MBT and monitor post-mitotic transcriptional reactivation.

Quantitative RNA detection methods suggest that transcriptional bursting is a key property of gene expression in diverse systems [90,91]. The term “bursting” refers to the episodic, discontinuous emergence of nascent transcripts at Pol II-bound loci. To investigate the links between enhancer control and transcriptional bursting at the MBT, *Fukaya* et al. placed well characterized enhancers upstream and downstream of reporter genes flanked by *MS2* and *PP7* stem loops [92]. They performed live-embryo imaging of the *MS2-yellow* reporter containing different *snail* (*sna*) enhancers of varying strength, along with its proximal promoter in different configurations. They observed major differences in bursting frequencies produced by the *sna* primary enhancer and by its shadow enhancer, a redundant regulatory sequence, which were correlated to the discrepancy in total RNA outputs. This analysis was extended to the *rhomboid* (*rho*), *Krüppel*; (*Kr*) and *Abdominal-B* (*Abd-B*) enhancers. By testing a set of conditions, Fukaya et al. showed that differential core promoter, distal enhancer and anteroposterior gradient positioning all affect bursting frequency, in line with discrepancies in total RNA outputs. Thus, Fukaya et al. identified the regulation of transcriptional bursting frequency as a key determinant of developmental gene activity at the MBT.

Puzzling disparities have long prevailed between reported rates of Pol II elongation (1.1–1.5 kb/min) and robust detection of several long de novo transcripts before the MBT [14,93]. Indeed, established elongation rates cannot account for the zygotic transcription of the 22 kb-long unit of Short gastrulation (*sog*) in NC13, when the time window permissive to transcription is narrowly restricted by a hasty interphase (10–12 min). Fukaya et al. solved this long-lasting paradox by revisiting Pol II elongation rates in early embryogenesis using dual-fluorescence through the MS2 imaging system [94] (Figure 7A). They measured an elongation rate of 2.4 kb/min, nearly twice that of previous estimates. This figure is compatible with endogenous *sog* transcription during the 13th interphase. In addition, they found that replacing the promoter or introducing a reporter containing an intron had little impact on elongation rate measurements, suggesting that elongation is not the rate-limiting step in transcription.

The inheritance of transcriptional states from mother to daughter cells, termed transcriptional memory, has been documented in the amoeba *Dictyostelium* [95,96]. In a recent study, Ferraro et al. monitored post-mitotic transcriptional reactivation of stochastically expressed transgenes using the MS2 imaging system [97]. This work provided the first evidence that transcriptional memory prevails at the massive wave of zygotic expression between the 13th and the 14th division. The authors used sensitized transgenes exhibiting patterns of sporadic expression to individually image the behavior of single cell lineages (Figure 7B). Daughter cells derived from nuclei that expressed the transgene during NC13, called memory mothers, were four times more likely to show early reactivation during NC14 interphase than daughters arising from non-memory mothers. Quantitative analyses of average fluorescence intensities revealed that memory nuclei produce, on average, two-fold more total mRNA than non-memory nuclei during NC14. These results provide strong evidence that transcriptional memory prevails during *Drosophila* MBT and impacts total RNA output, likely through modifications incurred at the level of nucleosomes, bound TFs or histone modifications following a first round of transcription. Ferraro et al. envision this emerging property of early transcription as a mechanism of developmental homeostasis, which could help ensure that cells retain the properties of their progenitors.

In brief, the recent deployment of in vivo imaging to document the transcriptional process in real time has revealed new insights into the dynamics of zygotic transcription. In vivo imaging has established the notion of transcriptional bursting, and shown that total gene-specific outputs at ZGA reflect the frequency of transcriptional bursts. It has enabled the revision of Pol II elongation rate at the MBT, reconciling the expression of lengthy genes such as *sog* with a short NC13 interphase. Quantitative imaging also showed that transcriptional memory prevails in *Drosophila* embryogenesis, promoting the rapid post-mitotic re-activation of sequences expressed during NC13, which likely contributes to developmental homeostasis.

## 7. Conclusions

In this review, we aimed to provide an overview of recent findings relevant to the transcriptome dynamics of early *Drosophila* development. Maternal control is essential in syncytial-stage embryos to sustain fast-paced proliferation in absence of a sizeable transcriptional output. Thus, we discussed the processes of RNA maternal deposition, localization and targeted clearance. Indeed, *Drosophila* embryos host a wealth of complex posttranscriptional regulatory processes. The spatiotemporal dosage of RBPs such as SMG reflect translational fine-tuning which, in turn, modulates the dynamics of hundreds of maternal mRNAs. RNA localization is highly prevalent in the large syncytial embryo and likely plays key roles in orchestrating the developmental program. Indeed, protein-coding transcripts adopt a large diversity of spatial distributions in early embryos, including subembryonic and exclusionary patterns, asymmetric anteroposterior localization and more resolved patterns such as membrane, microtubule or mitotic apparatus associations [24]. In many cases, the functional relevance of these mRNA localization events remains untapped, and awaits further characterization. Indeed, the fly model represents a powerful system to further dissect the *cis*- and *trans*-determinants regulating RNA localization events.

Maternal control is gradually met with an increasing contribution of the zygotic genome, as it progressively acquires transcriptional competence. The mechanisms accounting for ZGA onset are multifaceted and their underpinnings remain unclear. As the N/C ratio increases, a maternal factor responsible for transcriptional quiescence could be diluted against the mass of DNA, loosening the efficiency of the repression. Studies in *Xenopus* have identified maternal histone as putative transcriptional repressors [98]. In line with this finding, a recently identified linker H1 variant, dBigH1, has been associated to transcriptional quiescence. Indeed, dBigH1 is cleared out prior to the MBT and *big1* mutants show signs of disorganized chromatin and early zygotic transcription. Nevertheless, we know that the repressor titration model is not sufficient to account for the transcriptional silence of the zygotic genome in *Drosophila*. Indeed, most zygotic transcripts display similar expression kinetics in haploid and diploid embryos, which present very different N/C ratios [54]. This observation points to the molecular clock model, which proposes that egg activation sets a timer in motion to eventually trigger ZGA. One possible interpretation is that maternally contributed transcripts encoding pioneer TFs such as ZLD require time to be translated and accumulate sufficiently before pioneer activity has reached a level amenable to widespread ZGA. Together, a molecular clock set at egg activation and the rapid increase in nucleocytoplasmic ratio likely converge to exert changes in the structure of zygotic chromatin. Specific loci may exhibit enhanced sensitivity to pioneer TF activity. In addition, titration of the maternal repressor may not occur at a homogeneous rate across the genome. Such effects could account for the gradual nature of ZGA and explain why only a subset of zygotic genes display N/C-dependent expression dynamics.

Independently of its underlying mechanisms, once it has been triggered, zygotic transcription contributes to shaping a complex genome topology before the MBT, which largely remains in place throughout the life of the fly. After NC10, the growing population of transcripts produced by cortical nuclei play key roles in driving the course of development through the MBT. The transcriptional process itself exposes ssDNA and triggers the activation of the DNA replication checkpoint, possibly through the formation of stalled replication forks. Cellularization onset is linked to ZGA timing and it coincides with the loss of mitotic synchrony and the expression of additional zygotic genes. Real-time imaging has revealed that zygotic transcription proceeds as bursts, with the frequency of bursting events linked to its total RNA output. An example of transcriptional memory in *Drosophila*, the preferential post-mitotic reactivation of loci transcribed at NC13 has been demonstrated through this approach. In addition, embryonic Pol II elongation rates have been revised via the development of a dual fluorescence system. The deployment of real-time, in vivo RNA labeling to study transcription in *Drosophila* is still very recent. Future applications will likely contribute to clarify how ZGA is triggered. Indeed, important insights could be revealed by monitoring transcription in mutants of specific chromatin components, such as *bigH1*, or pioneer TFs, such as *zld*. This technology can notably reveal transcriptional dynamics at the single cell level, a sizeable advantage when investigating heterogeneous and multifaceted responses such as ZGA.
